# Glucose-6-Phosphate Dehydrogenase Deficiency and Haemoglobin Drop after Sulphadoxine-Pyrimethamine Use for Intermittent Preventive Treatment of Malaria during Pregnancy in Ghana – A Cohort Study

**DOI:** 10.1371/journal.pone.0136828

**Published:** 2015-09-01

**Authors:** Ruth Owusu, Kwaku Poku Asante, Emmanuel Mahama, Elizabeth Awini, Thomas Anyorigiya, David Dosoo, Alberta Amu, Gabriel Jakpa, Emmanuel Ofei, Sylvester Segbaya, Abraham Rexford Oduro, Margaret Gyapong, Abraham Hodgson, Constance Bart-Plange, Seth Owusu-Agyei

**Affiliations:** 1 Kintampo Health Research Centre, Ghana Health service, Kintampo, Ghana; 2 Dodowa Health Research Centre, Ghana Health Service, Dodowa, Ghana; 3 Navrongo Health Research Centre, Ghana Health Service, Navrongo, Ghana; 4 AngloGold Ashanti Malaria Control Program, Obuasi, Ghana; 5 Research and Development Division, Ghana Health Service, Headquarters, Accra, Ghana; 6 National Malaria Control Programme, Ghana Health Service, Korle-Bu, Accra, Ghana; Tulane University School of Public Health and Tropical Medicine, UNITED STATES

## Abstract

**Background:**

Sulphadoxine-Pyrimethamine (SP) is still the only recommended antimalarial for use in intermittent preventive treatment of malaria during pregnancy (IPTp) in some malaria endemic countries including Ghana. SP has the potential to cause acute haemolysis in G6PD deficient people resulting in significant haemoglobin (Hb) drop but there is limited data on post SP-IPTp Hb drop. This study determined the difference, if any in proportions of women with significant acute haemoglobin drop between G6PD normal, partial deficient and full deficient women after SP-IPTp.

**Methods and Findings:**

Prospectively, 1518 pregnant women who received SP for IPTp as part of their normal antenatal care were enrolled. Their G6PD status were determined at enrollment followed by assessments on days 3, 7,14 and 28 to document any adverse effects and changes in post-IPTp haemoglobin (Hb) levels. The three groups were comparable at baseline except for their mean Hb (10.3 g/dL for G6PD normal, 10.8 g/dL for G6PD partial deficient and 10.8 g/dL for G6PD full defect women).The prevalence of G6PD full defect was 2.3% and 17.0% for G6PD partial defect. There was no difference in the proportions with fractional Hb drop ≥ 20% as compared to their baseline value post SP-IPTp among the 3 groups on days 3, 7, 14. The G6PD full defect group had the highest median fractional drop at day 7. There was a weak negative correlation between G6PD activity and fractional Hb drop. There was no statistical difference between the three groups in the proportions of those who started the study with Hb ≥ 8g/dl whose Hb level subsequently fell below 8g/dl post-SP IPTp. No study participant required transfusion or hospitalization for severe anaemia.

**Conclusions:**

There was no significant difference between G6PD normal and deficient women in proportions with significant acute haemoglobin drop post SP-IPTp and lower G6PD enzyme activity was not strongly associated with significant acute drug-induced haemoglobin drop post SP-IPTp but a larger study is required to confirm consistency of findings.

## Introduction

The major adult risk group for malaria in endemic countries are pregnant women, especially primigravidae[1]^,^[2]^,^[3]. To prevent malaria in pregnancy, the World Health Organization (WHO) recommends the use of Sulphadoxine-Pyrimethamine (SP) for Intermittent Preventive Treatment in pregnancy (IPTp)[4] in addition to the use of insecticide treated nets (ITN’s) in areas of moderate-to-high transmission of malaria in Africa, as part of antenatal care services. SP is still the only anti-malarial recommended for IPTp. Intermittent Preventive Treatment of malaria in pregnancy using Sulphadoxine-Pyrimethamine (SP-IPTp) been shown to increase both maternal haemoglobin (Hb) at term and infant birth weight[5]^,^[6]^,^[7]. In Ghana, the National Malaria Control Programme started IPTp with SP in 2002 on a pilot basis in ante-natal clinics in some administrative districts. In 2005, this was rolled out to cover the whole country. SP was initially being given three times during pregnancy with at least one-month interval between doses but since early 2014, the country has implemented the current WHO recommendation of giving SP five times during pregnancy.

SP is a combination of Sulphadoxine (a sulphonamide) and Pyrimethamine, an anti-parasitic drug. Sulphonamides have generally been considered as one of the group of oxidant drugs which can cause haemolysis in G6PD deficiency[8]. Acute haemolytic anaemia is an uncommon complication with sulphadoxine which may be associated with G6PD deficiency or may be auto-immune mediated[9]. More recent studies have cited some sulphonamides as being contraindicated in G6PD deficiency because of the risk of haemolysis but have been silent on sulphadoxine [10]^,^[11]. SP has been found to decrease the level of reduced glutathione in the blood of rabbits and may thus induce oxidative stress by generating reactive oxygen species[12]. Even though drug metabolism in humans may not be the same as in rabbits, this is a very probable mechanism by which SP can cause oxidative drug-induced acute haemolysis in G6PD deficient individuals.

Glucose-6-phosphate dehydrogenase catalyzes the initial step in the pentose phosphate shunt, oxidizing glucose-6-phosphate to 6-phosphogluconolactone and reducing nicotinamide adenine dinucleotide phosphate (NADP) to the reduced form NADPH. NADPH acts as a cofactor in glutathione metabolism resulting in the production of reduced glutathione. Reduced glutathione is important in the removal of dangerous oxidative metabolites in the cell. In G6PD deficient individuals there is impairment of the G6PD dependent NADPH production and since the pentose phosphate pathway is the only source of NADPH in red cells, this results in impairment of production of reduced glutathione. This impaired production of reduced glutathione places patients with G6PD deficiency at increased risk for haemolytic anaemia when given oxidant drugs, because the red blood cells in these individuals are not able to handle the oxidative stress and consequently haemolysis ensues[11]^,^[13].

The few studies on G6PD deficiency among pregnant women in Ghana suggest a low prevalence of homozygous G6PD deficiency and a moderately high prevalence of heterozygous G6PD deficiency at least in parts of the country[14]. Even though heterozygous females usually have less severe manifestations some can develop severe drug–induced acute haemolysis when exposed to oxidant drugs leading to a significant haemoglobin drop[15]^,^[16]. The Anti-Malaria Drug Policy for Ghana recommends that pregnant women be screened for G6PD deficiency and those with G6PD deficiency be excluded from IPTp with SP. Despite the policy recommendation, most rural ante-natal clinics in Ghana do not have the capacity to determine G6PD status of pregnant women and majority of women attending antenatal clinics in rural areas do not know their G6PD status. This situation presents a dilemma to antenatal health care staff: whether to give SP to all eligible pregnant women in such areas without any consideration of G6PD status or to withhold SP. In most of such areas, SP is given to all pregnant women with unknown G6PD status who present at the antenatal clinics if they do not have any other contraindication to taking SP. Concerns have been raised that this practice may result in serious side effects including haemolysis, in G6PD deficient women who are unknowingly given SP. In Ghana, as in other parts of Africa, pregnant women are usually anaemic[17]^,^[18] and further decrease in the haemoglobin level of such women can be seriously detrimental. Even though preliminary results from post-administration surveillance during the pilot period of IPTp using SP indicated low level of adverse events[19]_,_ the surveillance did not specifically compare haemolysis in G6PD deficient women with G6PD normal women. The possibility of underreporting could also not be ruled out since the initial surveillance relied on self-reporting by the pregnant women[19]. Haemoglobin safety after SP use in G6PD deficient individuals has been poorly characterised. To date, no study in Ghana has compared haemolysis or haemoglobin drop in G6PD deficient women with G6PD normal women after SP use as IPTp.

In this study, we hypothesized that there was a difference between G6PD normal and G6PD deficient pregnant women in the proportion who develop significant acute haemoglobin drop post SP-IPTp. We quantified this difference between pregnant G6PD normal women and pregnant G6PD full deficient women and partial deficient women.

## Methods

### Study sites

**Fig 1:**
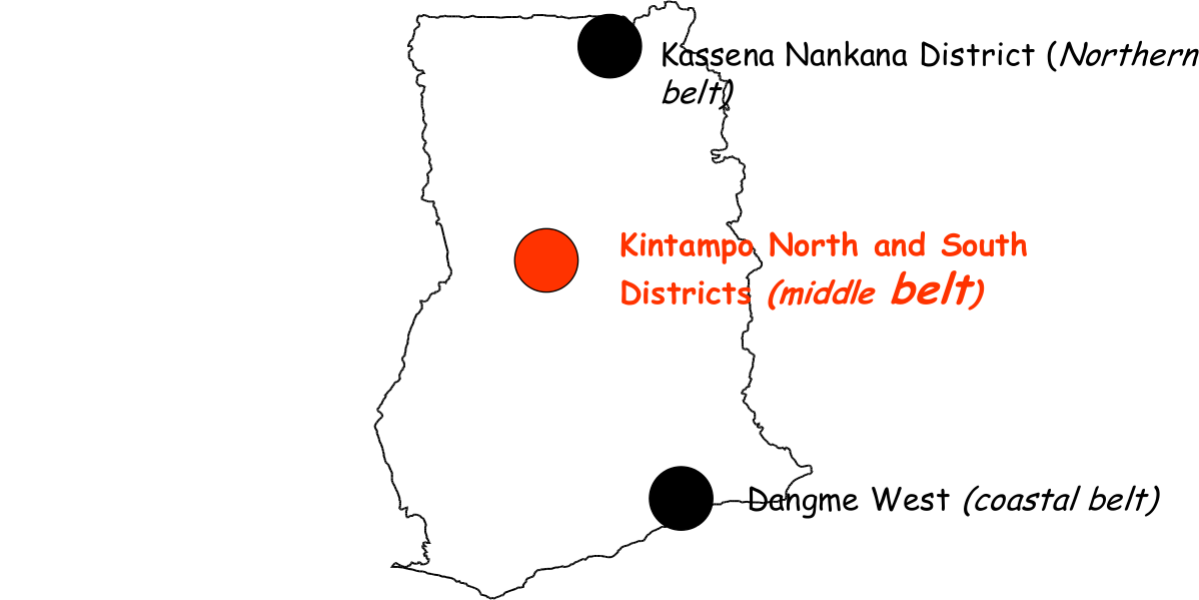
Map of Ghana showing the three study areas.

This was a nationally representative study involving three research centres under the Ghana Health Service. Each of these research institutions and their study sites are located within predominantly rural districts in one of the three main ecological zones in Ghana i.e. northern savannah belt (Kassena-Nankana district), the middle forest-savannah belt (Kintampo North and South districts) and the southern coastal belt (Dangme-West district) as shown in Fig 1. We therefore used pregnant women attending antenatal clinics in the three areas to represent the three main ecological zones of Ghana.

### Study population

Between June 2006 and April 2007, 1657 potentially eligible women were examined for eligibility. 1518 women were eligible and were all enrolled into the study. 502 of these were from the Kintampo North and South districts, 507 from the Kassena-Nankana district and 509 from the Dangme-West district.

### Study procedures and sampling methods

We purposively selected ante-natal clinics (ANC’s) within the study sites based on high attendance to enable recruitment within a reasonable time frame and eleven ANC’s in all participated in the study.

All pregnant women reporting to selected ANC’s were eligible to participate in the study if their pregnancy was between 16–32 weeks gestation and eligible to take SP-IPTp, resident in the study areas and gave witnessed informed consent. Women were excluded if they knew of or had previous reaction to sulpha-drugs, had history of having taken a sulpha-drug (including SP) within the last 4 weeks or had a malaria illness that required treatment. A pre-coded standardized questionnaire was used to collect basic demographic and obstetric information, past experience on SP intake and approximate date of conception of current pregnancy. Duration of gestation was assessed mainly by history of last menstrual period and examination of fundal height by an experienced midwife or clinician. Venous blood was taken for G6PD status determination, presence of malaria parasites, haemoglobin (Hb) levels and a liver function test (LFT). A urine specimen was also collected to determine baseline urine chemistry. Each pregnant woman received her SP IPT dose (3 tablets of: Sulphadoxine 500mg plus Pyrimethamine 25 mg provided) under direct observed therapy by ANC staff. The first day of enrolment into the study was classified as day 0.

### Follow-up of study participants

All participants were followed up by trained fieldworkers within 72 hours (day 3) after taking the SP treatment by a symptom assessment questionnaire to capture any adverse events experienced. Urine chemistry, capillary blood for Haemoglobin level and LFT were also done as part of the follow-ups. Study participants who complained of any illness were referred to see the study clinician for further management. They were again visited on days 7, 14 and 28 and interviewed for any possible reactions. Urine and blood samples were taken on these days as well.

### Laboratory methods

A G6PD quantitative assay was done (RANDOX, UK). G6PD activity was determined by measurement of the rate of absorbance change at 340 nm due to the reduction of NADP^+^. Results of the test were given as “G6PD Normal”, “G6PD Partial Defect” or “G6PD Full Defect”. Study participants were classified as: G6PD Normal if G6PD activity was ≥ 4000 mU/gHb (≥4.00 U/gHb), G6PD partial defect if G6PD activity was 1000–3999 mU/gHb (1.00–3.99 U/gHb) and as G6PD full defect if G6PD activity was < 1000 mU/gHb (<1.00 U/gHb).

The results of the G6PD test were written on each antenatal card at the end of the follow-up period or anytime a health worker requested before the follow-up period.

Haemoglobin was determined by means of the haemocuephotometer (Leo Diagnostics, Helsingborg, Sweden). The pregnant women were informed when their Hb readout was <6.0g/dL and treatment was initiated with ferrous sulfate tablets.

Serum alanine aminotransferase (ALT), aspartate aminotransferase (AST), total and direct bilirubin were done using a VitalabSelectra E Biochemistry analyzer (Vital Scientific, The Netherlands).

Malaria parasitaemia was determined by preparing thick and thin blood smears. Malaria parasites were counted against 200 white blood cells on the thick smear. A slide was declared negative only after 200 oil immersion fields of the thick smear had been examined. Parasites were expressed per microlitre, assuming a total white cell count of 8,000 per microlitre. Species identification was done using the thin smear. A sub-sample (10%) of the blood slides were randomly selected and sent to an independent microscopist not associated with the study for examination and the results compared with > 90% concordance.

### Sample size considerations

For the enrolled sample of 1216 G6PD normal, 255 G6PD partial defect and 36 G6PD full defect, with an expected 2% of G6PD normal, 6% of G6PD partial defect and 10% of G6PD full deficient developing the primary endpoint, we had 88% power to detect a difference between G6PD Normal and G6PD full deficient and 94% power to detect a difference between G6PD Normal and G6PD partial deficient but very low power (14%) to detect differences between G6PD partial defect and G6PD full defect women.

### Data Management and Analysis

All study questionnaires were reviewed and certified before being sent for data entry. Data were double-entered, verified and validated using Microsoft Access. Statistical analysis was carried out using Stata 11 (College Station, Texas, 7845, USA).

The main exposure variable was G6PD status, classified as G6PD Normal, G6PD partial defect (also referred to as partially deficient) and G6PD full defect (also referred to as full deficient). The main outcome variable was the proportion of study participants in each G6PD group with fractional Hb drop ≥ 20% (-0.20) on day 3 after SP intake.

Secondary outcome variables included proportion of study participants in each G6PD group with fractional Hb drop ≥ 20% (-0.20) on days 7, 14 and 28, correlation between G6PD activity and fractional Hb drop, and proportions of each G6PD group who had Hb > 8gdl at enrolment but who had their Hb dropping below 8 g/dl post-SP IPTp on days 3, 7, 14 and 28.

Fractional Hb drop on any follow-up day was calculated as the difference between the Hb on the follow-up day and Hb at enrolment (day 0 Hb) divided by the Day 0 Hb value i.e. the difference in Hb expressed as a fraction of the Day 0 Hb. A negative value indicated a drop in Hb at follow-up day as compared to the Day 0 Hb (this was sometimes expressed as a percentage in which case the negative value was not indicated).

Both descriptive and inferential analyses were performed. Point and interval estimates were computed. Frequencies and proportions of categorical variables were compared by Chi-square tests and continuous variables by one-way analysis of variance. P-values <0.05 were considered significant. Pearson's correlation was used to determine whether there was a linear relationship between G6PD activity and drop in haemoglobin. Correlation coefficients together with their corresponding p-values were estimated.

### Ethics Statement

Ethical approvals for the study were given by ethics review boards of the Ghana Health Service, the Kintampo Health Research Centre and the Navrongo Health Research Centre. Written informed consent was obtained from all women who participated in the study and participation was voluntary.The standard of care at the time of the study at the study sites, was not to test for G6PD deficiency prior to SP administration. G6PD testing in this study was done only after the standard antenatal care was provided. G6PD deficiency test results were however provided to the study participants and recorded in their antenatal card for their subsequent clinical management.

## Results

### Baseline demographic, obstetric and laboratory characteristics and G6PD prevalence

1657 potentially eligible women were examined for eligibility. 1518 women were eligible and were all enrolled into the study. 502 of these were from the Kintampo North and South districts, 507 from the Kassena-Nankana district and 509 from the Dangme-West district. G6PD results were available for 1507.

The overall prevalence of G6PD deficiency was 19.3% (291/1507); 2.3% G6PD full defect and 17.0% G6PD partial defect. The G6PD deficiency prevalence (partial and full defect) in the three ecological zones were similar; 21.8% (108/498) in the Kintampo Districts and 18.1% in both the Kassena-Nankana (91/502) and Dangme-West district (92/507).

Baseline demographic and obstetric characteristics are shown in Table 1 and were similar amongst the G6PD normal, G6PD partial deficient and G6PD full deficient women: mean age was 26.5 SD (5.9) in G6PD Normal, 26.7 SD (5.9) in G6PD partial deficient women and 26.5 SD (5.7) in G6PD full deficient women, mean gestational age 23.4 SD (4.7) weeks in G6PD normal women, 23.0 SD (4.8) weeks in G6PD partially deficient and 22.0 SD (4.6) weeks in the G6PD full deficient women, the majority with some level of formal education (61.4% in G6PD normal, 62.8% in G6PD partially deficient and 66.7% in G6PD fully deficient women) and proportion of primiparous women: 30.0% in G6PD Normal, 25.9% in G6PD partial deficient and 19.4% in G6PD full deficient women.

**Table 1:** Baseline demographic characteristics of study participants by G6PD status*.

Parameter	G6PD Normal N = 1216	G6PD partial deficient, N = 255	G6PD Full deficient N = 36	P-value
**Mean Age (SD)**	26.5 (5.9)	26.7 (5.9)	26.5 (5.7)	0.87
**Marital Status (n, %)**				
Married/Living together	1077 (88.57)	226 (88.63)	33 (91.67)	0.92
Widowed/Divorced/Separated/Never married	135 (11.10)	29 (11.37)	3 (8.33)	
Missing	4 (0.33)	0 (0.00)	0 (0.00)	
**Highest educational level (n, %)**				
None	469 (38.57)	95 (37.25)	12 (33.33)	0.08
Primary	271 (22.29)	46 (18.04)	5 (13.89)	
Secondary/Technical	443 (36.43)	111 (43.53)	16 (44.44)	
Post-Secondary/ Tertiary	29 (2.39)	3 (1.18)	3 (8.33)	
Missing	4 (0.33)	0 (0.00)	0 (0.00)	
**Parity (n, %)**				
1	365 (30.02)	66 (25.88)	7 (19.44)	0.58
2–5	743 (61.10)	165 (64.71)	25 (69.44)	
>5	84 (6.91)	16 (6.27)	3 (8.33)	
Missing	24 (1.97)	8 (3.14)	1 (2.78)	
**Mean Gestational age in weeks(SD)**	23.4 (4.7)	23.0 (4.8)	22.0 (4.6)	0.17

*G6PD status was available for 1507 study women.

Mean Haemoglobin (Hb) levels were significantly higher in G6PD full deficient (10.8 g/dl) and partial deficient women (10.8 g/dl) than in G6PD normal women (10.3 g/dl) at baseline (P<0.001). Geometric Mean (GM) Total bilirubin was also significantly higher in the G6PD deficient women than the G6PD normal women at baseline (P = 0.01). Proportions of the three groups with asymptomatic malaria parasitaemia at baseline were similar (10.3% for G6PD normal, 8.7% for G6PD partial defect and 11.4% in G6PD full defect, P = 0.73). Other baseline laboratory characteristics were similar among the three groups at enrolment as shown in Table 2.

**Table 2:** Baseline (Day 0) laboratory characteristics of study participants. (CI)* = 95% Confidence interval,

Parameter	G6PD Normal	G6PD partial defect	G6PD Full deficient	P-value
Mean Hb (SD) g/dl	10.3(1.54)	10.8 (1.40)	10.8 (1.46)	<0.001
Geometric Mean ALT (CI*)	9.0 (8.55–9.45)	8.5 (7.60–9.61)	10.1 (7.68–13.39)	0.516
Geometric Mean AST (CI*)	20.3 (19.72–20.95)	20.5 (18.84–22.43)	22.2 (18.34–26.90)	0.704
Geometric Mean total bilirubin (CI*)	4.9 (4.71–5.14)	5.4 (4.83–5.95)	7.4 (6.05–8.99)	0.005
Malaria Parasites present on blood smear (n, %)	124 (10.3)	22 (8.7)	4 (11.4)	0.733

### Follow-up

1239 (998 G6PD normal, 30 G6PD full defect and 211 G6PD partial defect) were available on the day 3 follow-up, 1269 (1021 G6PD normal, 33 G6PD full defect and 215 Partial defect) on day 7, 1215 (979 G6PD normal, 32 full defect and 204 partial defect) on day 14 and 1215 (980 G6PD normal, 33 full defect and 202 partial defect) on day 28 and were analysed.

### Mean Haemoglobin levels and drop in mean Haemoglobin

**Fig 2:**
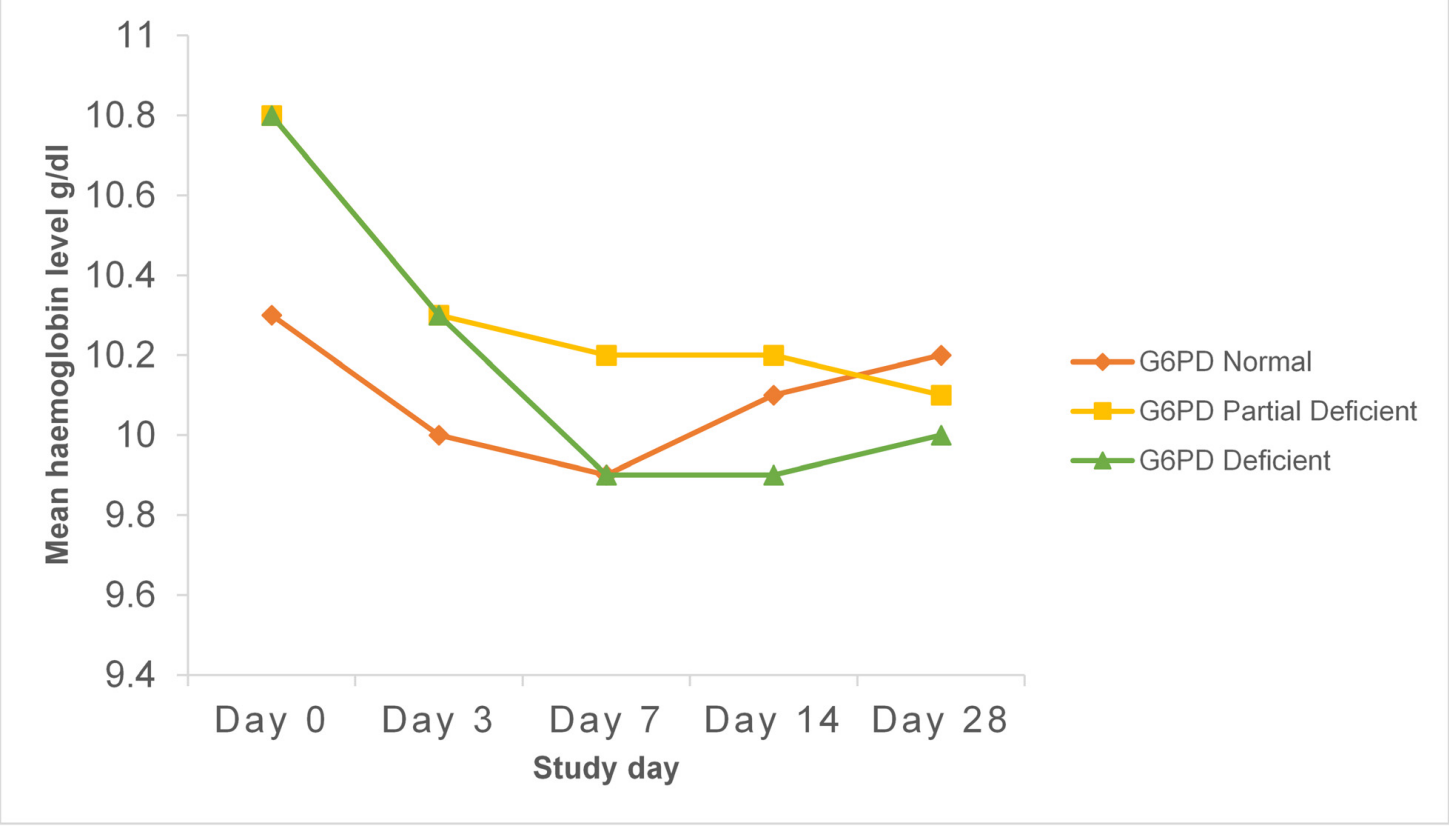
Mean Haemoglobin levels according to G6PD status and day of follow-up.

The mean haemoglobin levels on days 0, 3, 7, 14 and 28 are shown in Fig 2.

No study participant required blood transfusion or hospitalization for severe anaemia in this study.

Mean Haemoglobin concentration decreased from baseline in all three G6PD groups. The lowest mean Hb levels were recorded on day 7 for G6PD full defect (9.9 g/dl) and G6PD normal women (9.9 g/dl) and on day 28 for G6PD partial defect women (10.1 g/dl). There was a relatively higher decline in mean Hb for G6PD full deficient participants from day 0 to day 7 as compared to G6PD Normal and G6PD partial deficient women. The maximum decline in mean Hb was 0.4 g/dl for G6PD normal, 0.7 g/dl in G6PD partial defect and 0.9 g/dl for G6PD full defect women.

### Fractional haemoglobin drops by G6PD status on follow-up days

**Fig 3:**
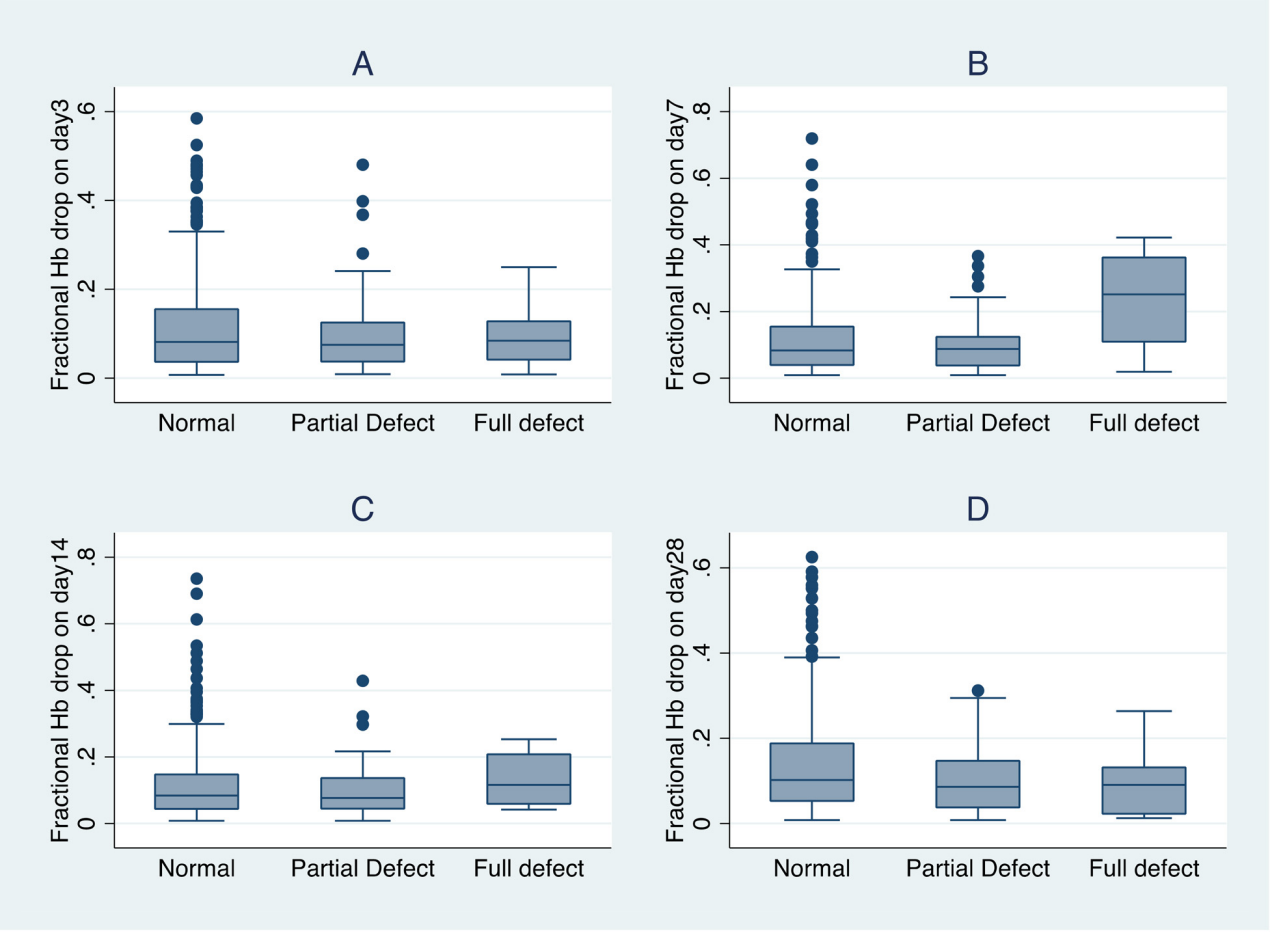
Box-plots for fractional Hb drops by G6PD status.

On days 3, 7, 14 and 28, the number of study participants who had a drop in their haemoglobin level as compared to their baseline were 482, 462, 463 and 526 respectively. Their fractional haemoglobin drops by G6PD status are described in Fig 3.

On day 3, median fractional Hb drops were similar for all three groups: -0.082 for G6PD normal, -0.075 for G6PD partial defect and -0.084 for G6PD deficient. On day 7, median fractional Hb drops for G6PD normal and G6PD partial defect increased slightly and were still similar: -0.083 and -0.087 respectively but the median fractional Hb drop for G6PD deficient increased sharply to -0.251. On day 14, median fractional Hb drops for G6PD normal and G6PD partial defect were almost similar to their day 3 value at -0.084 and -0.077 respectively and median fractional Hb drop for G6PD deficient was -0.116. On day 28, median fractional Hb drop for G6PD normal was the highest at -0.102, median fractional Hb drop for G6PD partial defect was -0.086 and median fractional Hb drop for G6PD deficient was -0.091.

### Fractional Hb drop ≥ 20% (-0.20) of baseline value

On days 3, 7, 14 similar proportions of G6PD normal, partial defect and full defect women had fractional Hb drop ≥ 20% as compared to their baseline value as can be seen in Table 3. On day 3, 7.21% (72/998) of G6PD Normal, 3.79% (8/211) of G6PD partial defect and 3.3% (1/30) of G6PD full defect had fractional Hb drop ≥ 20% of baseline (day 0) value, P = 0.145. On day 7, 4.83% (49/1021) of G6PD Normal, 5.12% (11/215) of G6PD partial defect and 9.09% (3/33) of G6PD full defect had fractional Hb drop ≥ 20% of baseline (day 0) value, P = 0.53. On day 14, 5.93% (58/978) of G6PD Normal, 2.45% (5/204) of G6PD partial defect and 6.25% (2/32) had fractional Hb drop ≥ 20% of baseline (day 0) value, P = 0.13. On day 28 however, the G6PD normal participants had a significantly higher proportion of women with fractional Hb drop ≥ 20% of baseline value. 10.21% (100/979) of G6PD normal, 5.45% (11/202) of partial defect and 3.03% (1/33) of G6PD full defect had fractional Hb drop ≥ 20% of baseline (day 0) value, P = 0.047

**Table 3:** Proportion of G6PD Normal, partial deficient and full deficient study participants with fractional Hb drop ≥ 20% (0.20) of baseline value.

Parameter	Hb drop ≥ 20% of baseline value		P-value
	Yes, n (%)	No, n (%)	
**Day 3 (N = 1239)**			
G6PD Normal	72 (7.21)	926 (92.79)	0.145
G6PD partial defect	8 (3.8)	203 (96.21)	
G6PD full defect	1 (3.3)	29 (96.67)	
**Day 7 (N = 1269)**			
G6PD Normal	49 (4.80)	972 (95.2)	0.532
G6PD partial defect	11 (5.12)	204 (94.88)	
G6PD full defect	3 (9.09)	30 (90.91)	
**Day 14 (N = 1214)**			
G6PD Normal	58 (5.93)	920 (94.07)	0.130
G6PD partial defect	5 (2.45)	199 (97.55)	
G6PD full defect	2 (6.25)	30 (96.9)	
**Day 28 (N = 1214)**			
G6PD Normal	100 (3.8)	892 (96.2)	0.047
G6PD Partial defect	11 (5.5)	188 (94.5)	
G6PD Full defect	1 (3.0)	32 (97.0)	

### Correlation between G6PD activity and fractional Haemoglobin drop

**Fig 4:**
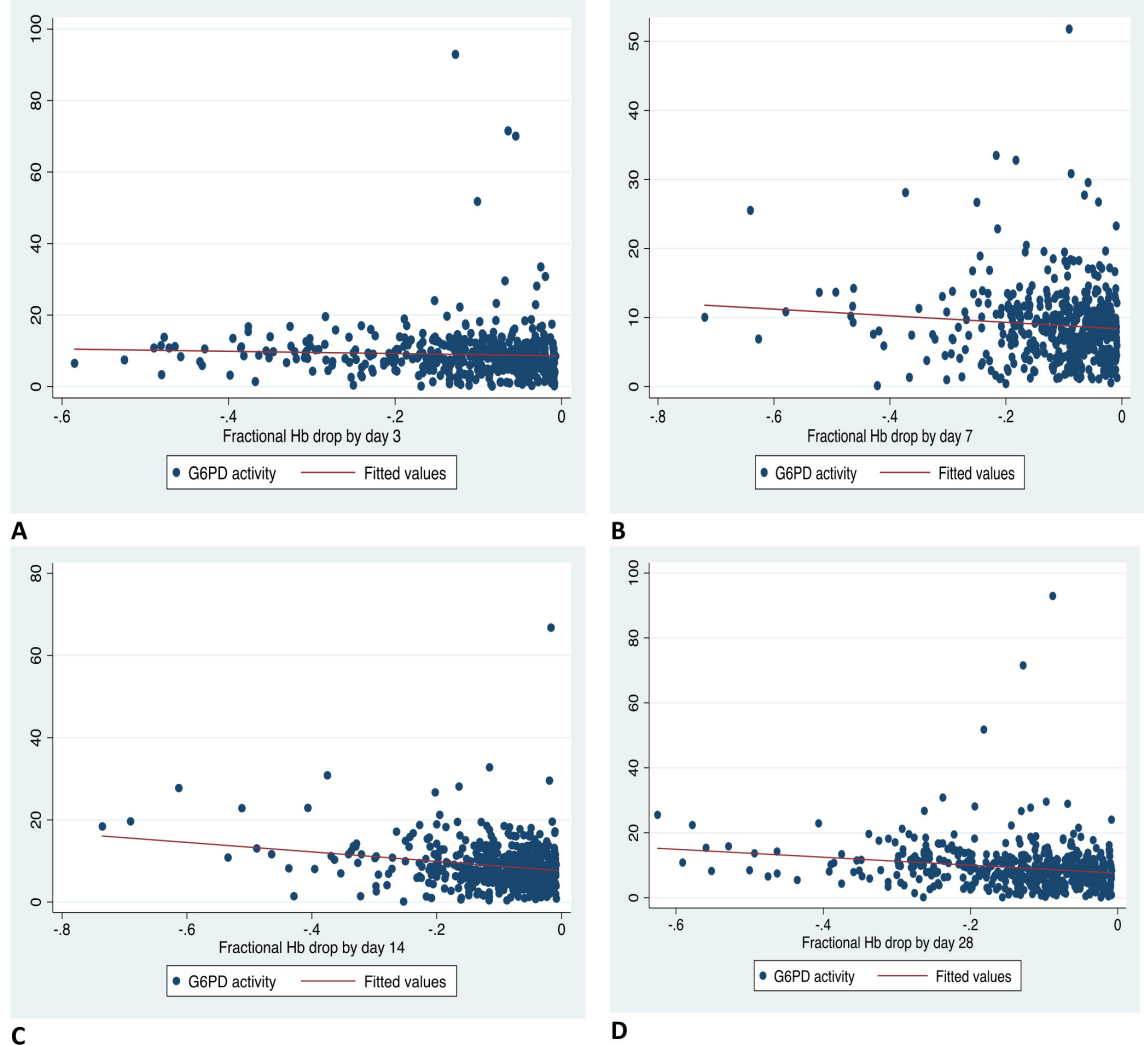
Correlation between G6PD activity (U/gHb) and fractional Hb drops in study participants with a decline in Hb from baseline post SP-IPTp.

Correlation between G6PD activity and fractional haemoglobin drop is shown in Fig 4. There was a very weak negative correlation between G6PD activity and fractional haemoglobin drop on days 3, 7, 14 and 28 which was statistically significant on days 14 and 28. On day 3, correlation coefficient (r) = -0.045, P = 0.324, (N = 482), on day 7, r = -0.066, P = 0.155, (N = 462), on day 14, r = -0.208, P<0.001 (N = 463) on day 28, N = 526, r = -0.186 and P<0.001.

**Fig 5:**
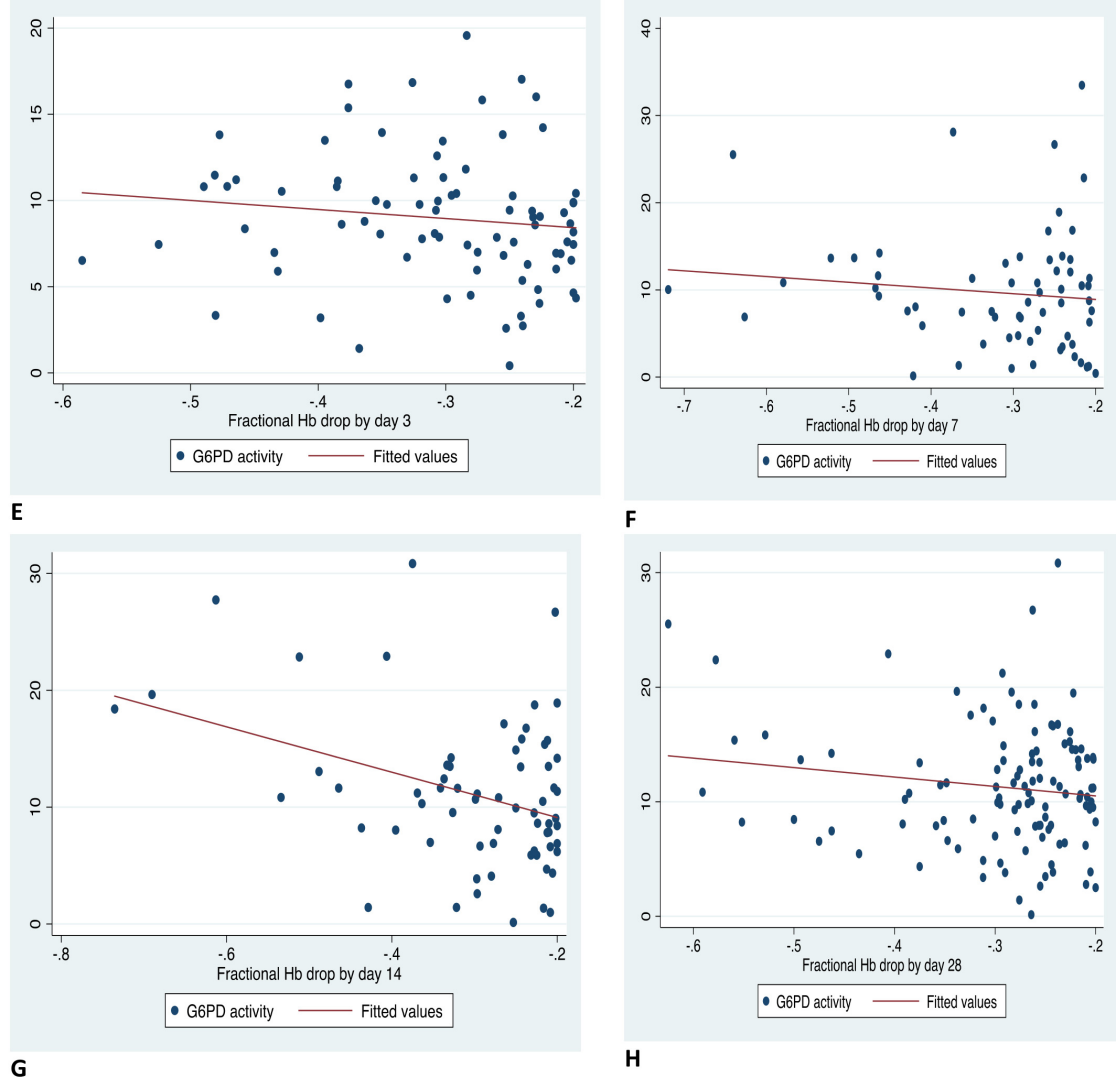
Correlation between G6PD activity (U/gHb) and fractional Hb drop in study participants with fractional Hb drops >20% (-0.20) post SP-IPTp as compared to baseline Hb.

When only those with fractional Hb drop > 20% (-0.20) were examined (Fig 5), G6PD enzyme activity was still weakly negatively correlated with fractional Hb drop which was significant only on day 14.

### Proportion of study participants with Hb ≥ 8g/dl on day 0 whose Hb level fell below the safety level of 8g/dl on subsequent follow-up days

On day of enrolment (day 0) 1434 study participants had Hb ≥8g/dl. Out of this sub-group, on day 3, 7.1% (67/943) of G6PD Normal, 4.8% (10/208) of G6PD partial defect and 6.7% of G6PD partial defect recorded Hb less than 8g/dl, P = 0.49 (as shown in Table 4). On day 7, 6.8% (66/967) of G6PD Normal, 5.7% (12/212) of G6PD partial defect and 6.1% (2/33) of G6PD. Full defect recorded Hb less than 8g/dl, P = 0.82. On day 14, 4.4% (43/979) of G6PD Normal, 3.0% (6/204) of G6PD partial defect and 3.1% (1/32) of G6PD full defect recorded Hb less than 8g/dl, P = 0.64. On day 28, 3.8% (32/927) of G6PD Normal, 5.5% of G6PD partial defect and 3.0% (1/33) of G6PD full defect recorded Hb less than 8g/dl, P = 0.50.

**Table 4:** Proportion of study participants with Hb ≥ 8g/dl on day 0 whose Hb fell below the safety level of 8g/dl in subsequent follow-up days.

Parameter	Hb<8g/dl	Hb≥8g/dl	P-value
**Day 3 (N = 1181)**			
G6PD Normal	67 (7.1)	876 (92.9)	0.487
G6PD partial defect	10 (4.8)	198 (95.2)	
G6PD full defect	2 (6.7)	28 (93.3)	
**Day 7 (N = 1212)**			
G6PD Normal	66 (6.8)	901 (93.2)	0.819
G6PD partial defect	12 (5.7)	200 (94.3)	
G6PD full defect	2 (6.1)	31 (93.9)	
**Day 14 (N = 1168)**			
G6PD Normal	41 (4.4)	894 (95.6)	0.636
G6PD partial defect	6 (3.0)	195 (97.0)	
G6PD full defect	1 (3.1)	31 (96.9)	
**Day 28 (N = 1159)**			
G6PD Normal	35 (3.8)	892 (96.2)	0.501
G6PD partial defect	11 (5.5)	188 (94.5)	
G6PD full defect	1 (3.0)	32 (97.0)	

There was no statistical difference between the three groups in the proportions of those who started the study with Hb ≥ 8g/dl whose haemoglobin level subsequently fell below the safety level of 8g/dl.

### Change in haemoglobin level for women with Hb less than 8g/dl at baseline

A subgroup analysis was carried out on 66 (62 G6PD normal, 4 Partial defect) women who had Hb < 8g/dl at day 0. When assessed on day 3, 31.0% (18/58) had a reduction in their Hb as compared to the baseline with the rest either having an increase or no change in Hb. The maximum decrease in Hb was on day 3 with 61.1% (11/18) having a drop less than 0.5 g/dl. Two women (both G6PD normal) had fractional Hb drop > 20% and had the highest Hb drops of 1.9 g/dl each: one of them with Hb of 7.5 g/dl at enrolment had Hb 5.6 g/dl on day 3 whilst another with Hb 7.7g/dl at enrolment had Hb 5.8 g/dl on day 3. By day 7, both women had an improvement in their Hb (as compared to the day 3 value) with Hb of 6.5 g/dl and 6.9 g/dl respectively and further improvement by day 28 with Hb of 7.4 g/dl and 7.6 g/dl respectively.

## Discussion

We examined the hypothesis that there is a difference in proportion of patients with significant acute haemoglobin drop after administration of SP as IPT among G6PD deficient pregnant women as compared with G6PD normal women. In G6PD deficiency, haemolysis may occur within six hours to three days of drug intake. The characteristic clinical and haematologic features include a drop in haemoglobin, haemoglobinuria, jaundice, hyperbilirubinaemia, heinz bodies in the peripheral red blood cells and a brisk reticulocyte response [13]^,^[20]. A significant drop in the haemoglobin levels of the person can occur within three days [11]^,^[13]. We used drop in haemoglobin levels (fractional haemoglobin drop) as a proxy for haemolysis in this study. Haemoglobin drop or change in haemoglobin concentration (either as absolute or fractional drop) has been the main outcome measure for a number of studies investigating the link between G6PD deficiency and post-drug administration haemolysis caused by oxidant anti-malarial drugs because changes in the haemoglobin level can be objectively and easily determined [21]^,^[22]^,^[23].

Contrary to our hypothesis, in this study, the proportion of women with fractional Hb drop > 20% (-0.20) as compared to the baseline within 3 days after intake of SP as IPT was similar among G6PD full deficient, G6PD partial deficient and G6PD normal women contrary to what we would expect if significant acute G6PD deficiency-associated haemolysis had occurred. The proportions with fractional Hb drop > 20% were similar among the study groups on days 7 and 14 as well. On day 28, the G6PD normal women had a significantly higher proportion with fractional Hb drop > 20% of baseline (day 0) value than the G6PD partial defect or G6PD full defect women (10.21%, 5.45% and 3.03% respectively, P = 0.047). In terms of fractional drop, however, this was not the whole picture: on days 3, 14 and 28 all three groups had similar median fractional Hb drop, but on day 7, the G6PD full deficient group had the greatest median fractional Hb drop of 0.251 (25.1%) and so even though the proportions of study participants who had fractional Hb drop > 0.20 (20%) were similar on day 7, the G6PD full defect women tended to have a greater fractional drop on day 7.

We also examined the proportion of study participants who started out with Hb ≥ 8g/dl who subsequently recorded Hb < 8g/dl. This end-point also captures proportion of study participants developing haemoglobin levels of potential safety concern even though it included some cases with very little reduction in their Hb. A safety level of 8 g/dl was chosen in consultation with practicing obstetricians. There was no statistical difference between the three groups in the proportions of those who started the study with Hb ≥ 8g/dl whose haemoglobin level subsequently fell below the safety level of 8 g/dl further strengthening the notion that there was not much difference between G6PD normal and G6PD deficient in terms of proportions of each group developing end-points of haemoglobin safety concern post SP-IPTp. Even among the women who were enrolled with low haemoglobin (Hb<8g/dl) none required blood transfusion and those who experienced a drop in Hb were in the minority and recovered well by day 28. It must be noted however that among this subgroup, none was G6PD full deficient and only a minority (6.1%) were G6PD partial deficient.

Drug-induced acute haemolytic anaemia caused by the antimalarial drugs Primaquine and Dapsone are two of the most studied G6PD deficiency-associated drug-induced acute haemolytic anaemia. The clinical and haematological course of drug-induced acute haemolytic anaemia in G6PD deficient individuals are strikingly similar: haemolysis starts within the first 24 hours with decrease in haemoglobin levels and the lowest haemoglobin levels usually occur at days 7 or 8 with recovery to baseline values between days 28 and 42[20]^,^[24]. In this study, mean haemoglobin concentration decreased from baseline in all three G6PD groups. The lowest mean Hb levels were recorded on day 7 for both G6PD full defect (9.9 g/dl) and G6PD normal women (9.9 g/dl) and on day 28 for G6PD partial defect women (10.1 g/dl). The maximum decline in mean Hb was 0.4 g/dl for G6PD normal, 0.7 g/dl in G6PD partial defect and 0.9 g/dl for G6PD full defect women. The haemoglobin levels of some of those who had a haemoglobin drop post SP-IPTp, did not fully recover to the baseline value by day 28 (in all 3 study groups) as can be seen from the day 28 median fractional Hb drops of -0.102 (10.2%), -0.086 (8.6%) and -0.091 (9.1%) for G6PD normal, G6PD partial defect and G6PD full deficient respectively. This may be due to pregnancy-related haemodilution but could possibly also be due to other known but uncommon adverse effects of sulphadoxine and pyrimethamine on bone marrow production especially in large doses[9]. It is quite unlikely, however, that relative overdosing could account for this. Patients received the standard dose of SP for a 60 kg adult i.e. a total dose of 1500 Sulphadoxine and 75mg Pyrimethamine (3 tablets of SP with 500mg Sulphadoxine and 25mg pyrimethamine each) as a directly observed treatment at the Antenatal Clinic. The therapeutic dose range for Sulphadoxine is 25mg–70mg /kg body weight and for Pyrimethamine it is 1.25 mg–3.25 mg/kg bodyweight. This means that this dose of SP can be safely given to those with much lower weights of even 25 kg and so even though we did not examine the relation between anthropometric measurements and fractional haemoglobin drop, relative overdosing is quite unlikely in this group of pregnant women in the second and third trimesters of pregnancy. Other studies which have investigated the long term effect of SP-IPTp on maternal anaemia have suggested an improvement in maternal anaemia after several doses of SP[5]^,^[7]^,^[25]. Even though none of these studies specifically investigated differences between G6PD normal and G6PD deficient women, SP for IPTp clearly is beneficial in the long term for both mother and baby[5]^,^[6]^,^[26] and as many pregnant women as can safely do so, should be encouraged to take SP as IPTp.

This study compared G6PD phenotype (determined using enzyme activity) with decline in haemoglobin levels post SP-IPTp. We determined G6PD activity using a quantitative spectrophotometric assay which is the gold standard for measuring G6PD activity[20]. In terms of determining haemoglobin safety in females when administering a potentially oxidant drug, G6PD phenotype based on enzyme activity is more relevant than G6PD genotype. In females, due to X chromosome inactivation, heterozygotes may have a wide range of enzyme activity, ranging from that similar to G6PD Normal to G6PD homozygote deficient and so simply knowing their genotype as heterozygote may not predict their residual enzyme activity and their reaction to oxidant drugs[20]. In this study there was a very weak negative correlation between quantitative G6PD activity and fractional Hb drop on all follow-up days which was significant only on days 14 and 28 (Fig 4). Decreasing G6PD enzyme activity was very weakly associated with higher haemoglobin drops and this was significant on days 14 and 28 only. This finding suggests lower G6PD enzyme activity is not associated with significant acute drug-induced haemoglobin drop post SP-IPTp.

Mean haemoglobin (Hb) levels at baseline were 10.8 g/dl in G6PD full deficient, 10.8 g/dl in G6PD partial deficient and 10.3g/dl in G6PD normal women. Even though mean haemoglobin (Hb) levels were statistically significantly higher in the G6PD deficient than G6PD normal at baseline (P<0.001), on the average, study women in both groups had mild anaemia and the 0.5 g/dl difference may not have much clinical significance. This finding of higher Hb at baseline in the G6PD deficient participants differs from a study in Nigeria among healthy participants in which the G6PD deficient participants had a lower haemoglobin at baseline which together with other findings led the authors to conclude that the G6PD deficient individuals had chronic subclinical haemolysis[27]. Our study may not be totally comparable to that study however since that study involved both females and males and this study involved pregnant women only and the G6PD activity thresholds used were different.

The updated WHO policy on IPTp does not require screening for G6PD deficiency but Ghana may have taken a more stringent approach because of reports of moderately high prevalence of G6PD deficiency among pregnant women[14]. The overall prevalence of G6PD deficiency (partial and full defect) in this study was 19.3% (2.3% full defect, 17% G6PD partial defect) and G6PD deficiency across the country was comparable as about the same proportions of women in the three main ecological zones (18–22%) were G6PD deficient. In the study mentioned above (by Mockenhaupt et al) which was carried out among a similar study population (pregnant women attending antenatal clinic in the Ashanti region of Ghana)[14], where the genotypes were determined, homozygous deficiency prevalence (2.6%) was similar to the 2.3% G6PD full deficiency in this study but the prevalence of heterozygous deficiency (30.4%) was quite different. It is not surprising that there is an apparent difference especially in heterozygous females because G6PD phenotype cannot be easily predicted from the genotype data especially in heterozygous females[28]^,^[29] and so the data of these two studies might still be compatible.

This study is subject to some limitations. The study was powered to detect differences between G6PD normal and G6PD partial defect and G6PD normal and G6PD full defect but not between G6PD partial defect and G6PD full defect and all results must be interpreted with this in mind. Another limitation of this study was that we studied the effect on haemoglobin levels after only one dose of SP, regardless of whether it was the first, second or third dose of IPTp and thus did not assess the safety of repeated doses. SP at the time of the study was given as three doses, one month apart from 16 to 32 weeks gestation in Ghana but since early 2014, the country has started implementing the current WHO recommendation of giving 5 doses[4]. It has been established by several studies that there is improvement in maternal anaemia at delivery after several doses of SP, [5]^,^[7]^,^[25] though none of these studies compared this outcome in relation to the G6PD status of the women. Assessing the safety of repeated doses in G6PD deficient women in this study would have required following up a larger sample size and following each study participant over at least four months together with the required laboratory testing. The funding available for the study was not adequate to cater for the related costs. However, given that there is paucity of research on this subject, we believe that our study, still provides relevant information on haemoglobin safety after SP-IPTp. In addition, there is current evidence that in individuals with G6PD A- which is the predominant variant in Sub-saharan Africa, new red cells formed after drug-induced haemolysis have relatively higher levels of G6PD activity and thus have decreased haemolysis with repeated doses of oxidant drugs[20].

The study results are not generalizable to pregnant women who are not indigenes of countries in Sub-Saharan Africa since they may have different G6PD variants.

## Conclusions

In Ghana, prevalence of G6PD full defect in pregnant women is low but G6PD partial defect prevalence is moderate. There was no significant difference between G6PD Normal and G6PD partial defect and G6PD full defect women in proportions with significant acute haemoglobin drop post SP-IPTp. Decreasing G6PD enzyme activity was weakly associated with higher haemoglobin drops, significant on days 14 and 28 only, suggesting lower G6PD enzyme activity was not strongly associated with significant acute drug-induced haemoglobin drop post SP-IPTp.

Though the findings from this study do not support G6PD deficient pregnant women being denied SP for IPTp, conducting a larger study will help to confirm consistency of our findings.
